# Engaging in cyber hygiene: the role of thoughtful decision-making and informational interventions

**DOI:** 10.3389/fpsyg.2024.1372681

**Published:** 2024-11-06

**Authors:** Christian Howell, David Maimon, Caitlyn Muniz, Eden Kamar, Tamar Berenblum

**Affiliations:** ^1^Department of Criminology, University of South Florida, Sarasota, FL, United States; ^2^Department of Criminal Justice and Criminology, Georgia State University, Atlanta, GA, United States; ^3^Department of Criminal Justice, The University of Texas at El Paso, El Paso, TX, United States; ^4^Institution of Criminology, Hebrew University of Jerusalem, Jerusalem, Israel

**Keywords:** cognition, cybersecurity, cyber hygiene, self-protection, rational choice, target hardening

## Abstract

**Introduction:**

The effectiveness of human-centric cybersecurity largely depends on end-users’ adherence to security and privacy behaviors. Understanding and predicting variations in the adoption of these safeguards is crucial for both theoretical advancement and practical application. While existing frameworks are often adapted from health science literature, there is potential to enhance these models by incorporating criminological constructs relevant to online victimization. This study introduce rational choice theory of thoughtfully reflective decision-making (TRDM) into the information security domain. TRDM suggests that variations in cognitive decision-making capabilities influence behavioral outcomes, particularly in the context of security and privacy practices.

**Methods:**

The study employed a field experiment to test the applicability of TRDM in predicting end-users’ engagement in security and privacy behaviors. Participants were exposed to security-related warnings, with the hypothesis that thoughtfully reflective decision-makers would be more likely to adopt robust protective behaviors. Data was collected on participants’ responses to these security warnings, as well as their overall adherence to privacy and security practices.

**Results:**

The findings support the theoretical framework: individuals exhibiting thoughtfully reflective decision-making tendencies demonstrated a higher likelihood of engaging in privacy and security behaviors. Specifically, participants with higher TRDM scores were more likely to adopt protective behaviors when warned of the consequences of non-compliance. These results indicate that cognitive decision-making capabilities significantly influence the likelihood of engaging in cybersecurity practices.

**Discussion:**

The study challenges the prevailing one-size-fits-all approach to cybersecurity by highlighting the importance of individual differences in cognitive decision-making. Thoughtfully reflective decision-makers are better equipped to adopt preventive security measures, suggesting the need for more tailored interventions in cybersecurity education and risk assessment. This research contributes to the development of sophisticated risk assessment tools aimed at mitigating vulnerabilities and reducing users’ susceptibility to digital threats. Incorporating TRDM into information security models provides a more nuanced understanding of user behavior, offering insights into how cognitive processes influence cybersecurity adherence.

## Introduction

As cybercrime victimization rates continue to rise (Hawdon, 2021), a simultaneous surge is observed in campaigns and initiatives dedicated to mitigating these escalating threats (Van Der Zee, 2021). Historically, efforts predominantly concentrated on developing software and services to safeguard internet users. However, a paradigm shift has transpired, driven by a confluence of factors—consumer reluctance to invest in additional protective services, skepticism about their effectiveness, and the recognition that traditional antivirus software inadequately shields against the full spectrum of cyber threats (Rainie, 2018).

In response to these challenges, organizations are increasingly turning to the proactive involvement of their employees and end-users (Kamar et al., 2022). This shift marks a departure from solely technological solutions, signaling a move toward human-centric cybersecurity. This evolution is embodied in the adoption of target hardening practices. Target hardening, as conceptualized by Clarke (1983), involves purposefully fortifying the security of potential targets by heightening the difficulty of committing crimes against them. In the context of traditional crimes, such as burglary, implementing target hardening strategies can be likened to installing additional lighting and a security system to deter potential intruders from gaining unauthorized access to a home.

In the digital realm, target hardening encompasses the integration of security protocols (e.g., strengthening passwords) and privacy precautions (e.g., limiting access to personal information) (Cain et al., 2018) as a comprehensive strategy to protect digital assets. The synergistic fusion of these elements encapsulates the essence of cyber hygiene, a term coined to represent the collective efforts aimed at maintaining a healthy and secure online environment (Vishwanath et al., 2020). For individuals to follow the best cybersecurity practices, they must understand the necessary actions and the implications of inappropriate behaviors. Beyond a theoretical framework, the effectiveness of cyber hygiene is supported by numerous studies across various disciplines (Ead and Abbassy, 2022; Maimon and Louderback, 2018; Singh et al., 2020). These studies consistently affirm the capability of cyber hygiene to not only mitigate but also prevent cyber-attacks.

Despite the established efficacy of target hardening in reducing susceptibility to victimization, it remains unclear why some individuals fail to adhere to these practices. Developing a theoretical model capable of predicting engagement in self-protection is an area of academic inquiry transcending subfield boundaries. The adoption of computer security and privacy behaviors, coupled with understanding victimization, are focal points in the information security and criminological literatures, respectively.

Since the decision to engage, or not engage, in self-protective behaviors temporally precedes victimization, a theoretical model could identify those most susceptible to victimization and encourage decisions ensuring their safety. Most studies concerning self-protection in cyberspace overlook criminological insights in favor of various theoretical models from the health sciences (Sommestad et al., 2015; Mou et al., 2022; Sulaiman et al., 2022). We argue that current explanations of engagement in cyber hygiene behaviors would significantly benefit from integrating criminological constructs, specifically those elucidating the concept of human agency—capturing the intentional alignment of actions with preferences for desired outcomes.

To address this gap, we introduce Paternoster and Pogarsky (2009) rational choice theory of cognition, known as thoughtfully reflective decision making (TRDM). Thoughtful decision making involves carefully considering and reconsidering the implications of one’s choices throughout the decision-making process to achieve the best possible outcomes. Paternoster and Pogarsky (2009) posit that variations in cognitive decision-making capabilities predict behavioral outcomes. Our study examines the role of TRDM in engagement in cyber hygiene behaviors, specifically its moderating role when individuals are exposed to the negative implications of not engaging in cyber hygiene. Thus, we focus on the cost aspect of the decision-making process, assuming that the permission request is illegitimate (i.e., from a malicious actor).

Our findings indicate that individuals endowed with higher cognitive decision-making capabilities, or thoughtfully reflective decision makers, are more inclined to adopt privacy behaviors due to their demonstrated efficacy in reducing online victimization. However, security practices were only adopted when thoughtfully reflective decision makers were exposed to educational content illustrating TRDM’s moderating role in the potential adverse outcomes of inadequate cyber hygiene. These results contribute to the expansion of criminological theory into information systems security, offering pivotal insights for the development of evidence-based crime prevention solutions.

## Literature review

### Situational crime prevention and target hardening

Situational crime prevention (SCP) scholars posit that offenders consciously make choices, and crime can be influenced by adjusting rewards and increasing associated consequences (Clarke, 1980). Cornish and Clarke (2003) identified five decision-influencing categories: (1) increase effort, (2) increase risks, (3) reduce rewards, (4) reduce provocations, and (5) remove excuses (Clarke, 1980, 1983, 1995; Cornish and Clarke, 2003). Within each category are five techniques designed to diminish the likelihood of criminal incidents (Cornish and Clarke, 2003). Among the twenty-five techniques spanning the five categories, Clarke (1983) emphasized the efficacy of “target hardening.” This method heightens the efforts required by motivated offenders to engage in criminal activities, as Clarke succinctly stated, ‘the most obvious way to reduce criminal opportunities is to obstruct or target harden’ (1983, p. 241).

Unlike most dispositional crime theories, SCP is particularly practical in reducing offending. Its versatile techniques are applicable to any crime in any setting, provided the prevention methods align with the specific situation (Clarke, 1995). When appropriately applied, SCP techniques, such as “target hardening,” are highly effective at decreasing criminal incidents (Clarke, 1995, p. 17).

In the physical world, diverse target hardening techniques effectively mitigate various crime types. For example, slug rejectors prevent slug use in parking meters and ticket machines (Decker, 1972; Clarke et al., 1994). Transparent barriers reduce assaults against bus drivers (Poyner, 1993) and diminish the number of robberies in post offices and banks (Ekblom, 1988; Clarke et al., 1991). Target hardening techniques, such as armored doors on airplanes, even contribute to a reduction in acts of terrorism (Clarke and Newman, 2006).

Given the success of target hardening techniques in reducing diverse forms of crime and the applicability of the SCP framework to various crime types across settings (Clarke, 1995), scholars argue that these techniques should play a role in reducing cybercrimes as well (Maimon and Louderback, 2018; Newman and Clarke, 2013).

Indeed, target hardening techniques have proven useful in preventing cyber-attacks against individuals (Levesque et al., 2013, 2016) and organizations (Back and LaPrade, 2020; Rege, 2014). At the organizational level, Rege (2014) found that amplifying security procedures, such as prevention and intrusion systems, reduces attacks on power grids. At the individual level, engaging in recommended security behaviors can reduce various forms of victimization, including password cracking, computer infection, data loss, and hacking victimization (Weir et al., 2010; Choi, 2008; Levesque et al., 2013, 2016; Wilsem, 2013). Levesque et al. (2013, 2016) conducted clinical trials assessing the effectiveness of antivirus software in detecting and preventing computer infections on personal devices. The studies revealed that nearly 50% of devices would have been infected without antivirus software, demonstrating the utility of certain target-hardening behaviors in cyberspace.

### Cyber hygiene

No singular technique can prevent all forms of online victimization. For instance, antivirus software is effective against computer infection (Levesque et al., 2013, 2016), while a robust password is necessary to thwart brute force attacks (Weir et al., 2010). Self-protection in cyberspace demands adherence to a variety of target hardening techniques, collectively known as cyber hygiene (Cain et al., 2018).

The term “cyber hygiene” gained prominence during the 2014 National Campaign for Cyber Hygiene, organized by the Center for Internet Security (CIS) and the Governors Homeland Security Advisors Council (GHSAC) (Maennel et al., 2018). This campaign likened cyber hygiene to personal hygiene, suggesting that preventative measures can mitigate cybercrime incidents, akin to how hand washing prevents the spread of disease. Emphasizing increased awareness of risks associated with poor cyber hygiene, the campaign aimed to boost adherence.

According to Maennel et al. (2018) formal definition (2018, p.1), cyber hygiene is defined as ‘a set of practices aiming to protect from negative impact to the assets from cyber security related risks.’ This definition underscores the human factor in risk reduction, requiring internet users to actively engage in routine preventative behaviors tailored to their security needs.

The present study focuses on individual-level cyber hygiene, where it encompasses both security and privacy practices. Security behaviors strategically fortify networked devices, such as the installation of antivirus software and the use of complex passwords. Privacy behaviors aim to limit personal information collection by thoughtfully managing online behaviors, privacy settings, and information sharing.

In a recent study, Howell (2021) demonstrated that heightened engagement in cyber hygiene practices aligns with the concept of target hardening in the cyber domain, supporting the principles of the SCP framework. Howell’s findings highlight that increased cyber hygiene directly correlates with a reduction in online victimization, emphasizing the effectiveness of proactive self-protective measures.

Yet, the motivations behind individuals abstaining from adopting self-protective measures, like cyber hygiene, despite their potential to enhance target hardness, remain unclear. In this study, we utilize Paternoster and Pogarsky (2009) rational choice theory of cognition, known as TRDM, to pinpoint those most susceptible to cyber victimization.

### Thoughtfully reflective decision making (TRDM)

The rational choice approach is grounded in the assumption of human agency (McCarthy, 2002). Consequently, scholars within this framework view individuals not only as decision-makers but also as agents who impose their choices on the world (Nagin, 2007). Decisions are deemed rational when they align with the decision maker’s preferences for outcomes (McCarthy, 2002; Nagin, 2007; Paternoster and Pogarsky, 2009). However, it’s crucial to acknowledge that not all individuals possess equal capabilities to make decisions in line with their preferences. As pointed out by Paternoster and Pogarsky (2009, p. 104), “On average, some persons are better than others at collecting information or collecting more or better information; they are more careful in weighing the costs and benefits, more thoughtful in considering the information gathered, and more likely to ask themselves later if they could have made a better decision.”

Recognizing that not all actions are inherently rational (McCarthy, 2002) and that individuals differ in their ability to make decisions leading to favorable outcomes (Baron, 2009), Paternoster and Pogarsky (2009) introduced TRDM, a rational choice theory of cognition. According to the theorists, thoughtfully reflective decision makers exhibit intentionality (i.e., collecting information pertinent to the problem), forethought (i.e., thinking of alternative solutions to the problem), self-reactiveness (i.e., systematically deliberating over how to determine which alternative might be best), and self-reflectiveness (i.e., retrospectively analyzing how good a problem solver one was in the situation). Therefore, TRDM encapsulates the essence of human agency, illustrating the process of reasoned decision-making most likely to yield the intended outcome.

Paternoster and Pogarsky (2009) argued that individuals with higher TRDM should exhibit more successful life outcomes and a reduced risk of antisocial behavior. Empirical research generally supports this proposition, establishing TRDM’s predictive power over both prosocial and antisocial behavioral patterns (Maimon et al., 2012; Paternoster and Pogarsky, 2009; Paternoster et al., 2011; Timmer et al., 2020).

The application of TRDM to victimization and the cyber-environment is limited. Louderback and Antonaccio (2017), using survey data gathered from a large private university, examined the relationship between TRDM and criminal behavior in the cyber-environment. Their findings revealed that thoughtfully reflective decision makers were less likely to engage in, or fall victim to, cybercrime incidents than their counterparts. The effect of TRDM on victimization demonstrates that quality decision making is pertinent to the discussion of self-protection. Although untested, the authors believed the observed effect between TRDM and online victimization occurred because those with lower levels of TRDM “are less likely to engage in thoughtful cognitive decision-making processes when taking steps to protect their computers against potential victimization” (Louderback and Antonaccio, 2017, p. 645). The current study seeks to test this assertion using a field experiment.

### Current study

Given that cyber hygiene has been shown to reduce victimization experiences (Howell, 2021), and recognizing that thoughtfully reflective decision makers tend to make decisions leading to conventionally better life outcomes (Paternoster et al., 2011), we propose the following hypothesis:

1. Hypothesis 1: Thoughtfully reflective decision makers are more likely to engage in cyber hygiene behaviors.

Additionally, Paternoster and Pogarsky (2009) assert that TRDM is dynamic, indicating that decision-making varies across contexts and can be improved through targeted educational efforts. Education plays a significant role in altering the decision-making process by providing additional information that assists decision makers in aligning their choices with their preferences for outcomes. Furthermore, thoughtfully reflective decision makers, on average, possess better capabilities to make quality decisions due to their enhanced ability to consider relevant information, including that provided through educational programming. Consequently, when presented with educational content outlining potential negative outcomes associated with a particular course of action, thoughtfully reflective decision makers are more likely than their counterparts to opt for an alternative course of action to improve the anticipated outcome (Paternoster et al., 2011). Building on this, we propose the following hypothesis:

2. Hypothesis 2: Thoughtfully reflective decision makers, when warned about the negative implications of failing to engage in cyber hygiene behaviors through educational information, are more likely to participate in cyber hygiene behaviors.

## Materials and methods

Data for the present study were gathered In Israel in November 2016 by administering an online survey followed by online field experiment examining both components of cyber hygiene: privacy and security. The survey and experiments were conducted using iPanel, an Israeli Internet panel service. iPanel stands as a premier provider of an extensive range of online data collection services. Within its offerings, the company manages the largest and most comprehensive online panel in Israel, boasting a membership exceeding 100,000 individuals aged 12 and older. This service provides a representative sample of Israeli Internet users by logging and tracking approximately 100 demographic, psychographic, and consumer data points for each respondent. Out of the total number of iPanel members, 164 agreed to participate in the study. After participation, respondents were rewarded with survey credits that could be converted to gift vouchers. The Institutional Review Board approved all study procedures.

The final sample size of *N* = 82 for each context represents the number of individuals who agreed to participate in the study. We conducted a power analysis to determine the required sample size for a moderate effect size, with an odds ratio of 2.48, corresponding to a Cox index of 0.3 to 0.5, a significance level of *p* = 0.05, and a desired statistical power of 0.80 without other predictors included in the analysis (*R*^2^ = 0). The power analysis determined that a sample size of 71 participants would be required to achieve the desired power. However, it is important to note that we included control variables in our analysis, which slightly adjusts the baseline number of required participants. While binary logistic regression (BLR) typically necessitates a larger sample size than multiple linear regression (MLR) due to the dichotomous nature of the data, our sample size remains adequate for detecting moderate effect sizes. Additionally, any potential issues with statistical power would likely deflate our findings rather than inflate them, ensuring a conservative interpretation of the results.

### Procedure

Participation was allowed using cellular phone browsers only, and those who had previously participated in the study were blocked using a cookie. Participants were first presented with a questionnaire consisting of a standard informed consent acknowledgment, questions regarding demographic characteristics (i.e., age, gender, and education level), questions aimed to assess cognitive decision-making capabilities as measured by TRDM (Paternoster and Pogarsky, 2009), and questions regarding the participant’s familiarity with computers.

After completing the questionnaire, participants were randomly assigned to the privacy or security dimension assessment. Participants were then presented with five cognitive task blocks in a randomized order to provide stimuli for facilitating the study. The cognitive tasks used in the study were: a flanker task (Eriksen and Eriksen, 1974), a Stroop task (Stroop, 1935), a global-local task (Navon, 1977), a conjunction-search task (Treisman and Gelade, 1980), and a Simon task (Simon, 1969). The cognitive tasks were solely used to disguise the study’s objectives and facilitate progress in the application. Therefore, the responses to these cognitive tasks were neither recorded nor analyzed. During the completion of the cognitive tasks, participants encountered pop-up requests for either privacy or security permissions, allowing assessment of cyber hygiene behavior.

### Privacy behavior

Participants who were assigned to the privacy dimension (*n* = 82) were randomly assigned to the treatment (*n* = 42) and control (*n* = 40) groups. Participants in the treatment group were presented with privacy permission requests accompanied by educational information explaining the implications of accepting the requests. Whereas participants in the control group were only presented with privacy permission requests. The exact text of permission requests for the privacy study is presented in Table 1.

**TABLE 1:** Privacy Compromising Requests.

Short version (Control) Explanation-absent	Long version (Treatment) Explanation-present
The site is requesting access to your Facebook profile.	The site is requesting access to your Facebook profile. Accessing your Facebook profile allows exposure to your friends list.
The site is attempting to access your Google search history.	The site is attempting to access your Google search history. Google history includes all your previous searches. Accessing your Google search history exposes personal information.
The site is requesting access to your current location.	The site is requesting access to your current location. Accessing your location services allows the site to follow your location.
The site is attempting to access the device’s surfing history.	The site is attempting to access the device’s surfing history. Accessing your surfing history exposes your habits regarding surfing from the device.

### Security behavior

Participants (*n* = 82) who were assigned to the security dimension were randomly allocated to the treatment (*n* = 42) and control (*n* = 40) groups. Participants assigned to the treatment group were presented with security permission requests accompanied by educational information explaining the implications of accepting the requests, while the control group was only presented with permission requests. The exact text of the security requests is detailed in Table 2.

**TABLE 2:** Security compromising requests.

Short version (Control) Explanation-absent	Long version (Treatment) Explanation-present
The site is attempting to open a port in your firewall	The site is attempting to open a port in your firewall. Opening a port might allow viruses’ access to your device.
The site is attempting to run Java script.	The site is attempting to run Java script. Running Java scripts might damage your device.
The site is attempting to install a VPN profile.	The site is attempting to install a VPN profile. Installing a VPN profile will expose your device to access by other devices.
The site is attempting to access your browser’s cookies.	The site is attempting to access your browser’s cookies. Accessing cookies allows following your surfing habits from this device.

### Dependent variable

Importantly, and as discussed in depth above, cyber hygiene consists of both security and privacy behaviors. Once a third party has access to personal information, it can be monetized or used to aid in further victimization. For example, geolocation assists stalkers in locating their victim. Moreover, personal information ascertained from a Facebook account or search history can be used to develop effective spear-phishing messages. Privacy behaviors thus include behaviors aimed at restricting the way in which personal information is collected and used. For Study 1, adherence to privacy behaviors was operationalized as denying access to the following: Facebook profile, Google search history, location services, and surfing history.

Conversely, security behaviors include any behavior aimed at protecting networked devices from being compromised. For Study 2, adherence to security behaviors was operationalized as denying access to the following requests: open a port in their firewall, run Javascript, install a VPN profile, and access their browser’s cookies. Granting access to any of these requests puts the device at risk of being compromised.

For either study, once a participant denied access to *any* of the requests, the study was concluded. Therefore, if the participant denied access to at least one of the four presented requests they were coded as 1. If they did not deny access to at least one of these requests, they were coded as 0. Thus, our dependent variable, *Deny Access*, is a binary variable wherein 1 represents engaging in self-protection in the form of privacy (Study 1) or security (Study 2). Importantly, this method of data collection expands upon prior research by assessing real, rather than perceived, behavior.

### Independent and control variables

Educational information explaining the *Implication (of) Disclosure* reflects subjects’ random assignment to treatment and control groups. Participants in the treatment group received an explanation of the possible implications associated with the request, whereas those in the control group did not. Those who received the explanation were coded as 1, those who did not were coded as 0.

*Thoughtfully Reflective Decision Making (TRDM)* was measured based on Paternoster and Pogarsky (2009) four-item scale. Respondents were asked to rank their agreement (1 “Strongly Disagree” to 5 “Strongly agree”) with the following statements: “When I have a problem to solve, one of the first things I do is get as many facts about the problem as possible,” “When I am attempting to find a solution to a problem, I usually try to think of as many different approaches to the problem as possible,” “When making decisions, I generally use a systematic method for judging and comparing alternatives,” “After carrying out a solution to a problem, I usually try to analyze what went right and what went wrong.” Scores were then summed to create a TRDM scale with higher scores indicating more reflective decision-making (Paternoster and Pogarsky, 2009). We then centered the TRDM scale to the mean.

*Operational Internet Tasks* was based on van Deursen et al. (2014) measures and was assessed using a two-item composite scale. Respondents were asked to rank how often they engage with the following tasks online (1 “Never” to 5 “Daily”): “upload files to another computer” and “download programs.” Scores were then summed to create a scale ranging from 2 to 10 with higher scores indicating that the person engages in more Operational Internet Tasks.

*Information Internet Tasks* was also based on van Deursen et al. (2014) measures and was assessed using a three-item composite scale. Respondents were asked to rank how often they engage with the following tasks online (1 ‘Never’ to 5 ‘Daily’): “make a decision based on retrieved information,” “use advanced search options,” and “find the information you were looking for.” Scores were then summed to create a scale ranging from 3 to 15 with higher scores indicating that the person engages in more Information Internet Tasks.

We also included gender, age, and education as control variables. *Male* was coded as 1 if the respondent identified as male. To measure *age*, respondents were simply asked to self-report their age in years. *Education* was coded as an ordinal variable ranging from 1 (elementary education) to 6 (advanced academic degree). Given the homogeneity of the Israeli population, we did not control for race.

## Results

### Study 1. Effect of TRDM on privacy behaviors

After generating descriptive statistics for the study sample, we used logistic regression to test our first hypothesis and determine whether *TRDM* is related to denying access to privacy compromising requests. Then, we created an interaction term between *TRDM* and *Implication of Disclosure* to test our second hypothesis. Descriptive statistics are reported in Table 3. All utilized scales had acceptable internal reliability (alpha > 0.70).

**TABLE 3:** Descriptive Statistics.

	Privacy behavior (*n* = 82)				Security behavior (*n* = 82)			
	Mean/% (*n*)	SD	Min	Max	Mean/%/ (*n*)	SD	Min	Max
Deny Access	26% (21)	0.44	0	1	33% (27)	0.47	0	1
Implication of Disclosure	51% (42)	0.50	0	1	51% (42)	0.50	0	1
TRDM	14.88	3.10	4	20	14.51	3.48	4	20
Age	36.00	13.04	18	70	34.39	11.72	18	69
Male	38% (31)	0.49	0	1	39% (32)	0.49	0	1
Education	4.37	1.17	1	6	4.46	1.07	1	6
Information Internet Tasks	4.33	0.82	1	5	4.21	1.04	1	5
Operational Internet Tasks	2.40	1.19	1	5	2.51	1.08	1	5

Interestingly, only 26% of respondents denied one of the application requests for access to their private information. Additionally, although participants differed in their level of *TRDM*, with scores ranging from 4 to 20, the average score was high (M = 14.88; SD = 3.10). The average score for *Operational Internet Tasks* was somewhat low (M = 2.40; SD = 1.19), while the average score for *Information Internet Tasks* was relatively high (M = 4.33; SD = 0.82). Finally, the average age of participants was 36 (SD = 13.04), most respondents identified as female (*n* = 51), and most respondents were college educated (M = 4.37; SD = 1.17).

Results of the logistic regression analyses are presented in Table 4. Model 1 shows that individuals with higher levels of *TRDM* were more likely to deny access when asked for access to private information (*b* = 0.23, *p* = 0.04). In other words, and in support of hypothesis 1, thoughtfully reflective decision makers were more likely to engage in online self-protection through denying requests that would infringe upon their privacy. In addition to *TRDM*, we found that individuals with higher levels of education were also more likely to deny requests that infringe upon their privacy. Specifically, *Education* was associated with a nearly 400% increase in the odds of denying access (*b* = 1.60, *p* = 0.02). In contrast to expectations, *Information Internet Tasks* were associated with a decreased odds of denying access (*b* = −1.17, *p* = 0.02).

**TABLE 4:** Logistic Regression Estimating Effect of TRDM on Privacy Behaviors (*n* = 82).

	Model 1		Model 2	
	B(SE)	OR	B(SE)	OR
Implication Disclosure	−0.24(0.64)	0.78	0.58(3.17)	1.79
Mean-Centered TRDM	0.23(0.13)	1.26*	0.26(0.19)	1.30
Age	−0.16(0.11)	0.85	−0.16(0.11)	0.85
Male	−0.68(0.64)	0.50	−0.66(0.64)	0.51
Education	1.60(0.73)	4.98*	1.58(0.73)	4.87*
Information Internet Tasks	−1.17(0.53)	0.31*	−1.18(0.54)	0.31*
Operational Internet Tasks	0.16 (0.23)	1.18	0.17(0.23)	1.19
Implication of Disclosure*TRDM	−	−	−0.05(0.20)	0.95
Constant	−5.49 (2.03)	0.12	−6.85(1.73)	0.09
Pseudo *R* square Log Likelihood	0.37 −29.00		0.38 −28.97	

The *R*^2^ value reported is the Nagerkerke *R*^2^.

*p < 0.05 (one-tailed).

The results of Model 2, which included an interaction term for *TRDM* and *Implications of Disclosure*, were similar to Model 1. The only notable difference was a loss in significance for the *TRDM* variable. The only notable difference was a loss in significance for the *TRDM* variable. In contrast to our second hypothesis, thoughtfully reflective decision makers, who were warned of the negative implications of not engaging in cyber hygiene behaviors, were *not* more likely to engage in cyber hygiene behaviors. This is likely because they did not need the additional information to make the decision to engage in privacy behaviors. In essence, since thoughtfully reflective decision makers typically consider all potential outcomes, the additional information about the negative consequences of disregarding privacy behavior might have overwhelmed them with information, leading to a less favorable outcome.

Interestingly, being warned of the negative implications associated with allowing access to private information was not significantly associated with an individual’s privacy behavior in either model. Furthermore, in neither model were respondents’ *Age, Gender*, nor *Operational Internet Tasks* statistically significant.

### Study 2. Effect of TRDM on security behaviors

After generating descriptive statistics for the study sample, we used logistic regression to further test our first hypothesis and determine whether *TRDM* is related to denying access to requests that comprise the security of the device. Then, we created an interaction term between *TRDM* and *Implication of Disclosure* to test our second hypothesis. Descriptive statistics are reported in Table 3 and resemble the descriptive statistics of Privacy behavior assessment. All utilized scales had acceptable internal reliability (alpha > 0.70).

Here, only 33% of respondents denied one of the application requests. Again, participants differed in their level of *TRDM*, with a rather high average score (M = 14.51; SD = 3.48). The average score for *Operational Internet Tasks* was somewhat low (M = 2.51; SD = 1.08), while the average score for *Information Internet Tasks* was relatively high (M = 4.21; SD = 1.04). Finally, the average age of participants was 34 (SD = 11.72), most respondents identified as female (*n* = 50), and most respondents were college educated (M = 4.46; SD = 1.07).

Results of the logistic regression analyses are presented in Table 5. Findings, depicted in Table 5, Model 1, reveal that *TRDM* was *not* a significant predictor of engaging in security behaviors. This is in direct opposition to our first hypothesis. Interestingly, only *Age* was associated with self-protection in the form of denying access to security compromising requests. Specifically, older individuals were less likely to deny access to our requests to compromise the security of their device (*b* = −0.09, *p* = 0.004).

**TABLE 5:** Logistic Regression Estimating Effect of TRDM on Security Behaviors (*n* = 82).

	Model 1		Model 2	
	B(SE)	OR	B(SE)	OR
Implication of Disclosure	−0.21(.52)	0.81	−6.29(2.42)	0.01*
Mean- Centered TRDM	−0.08(.07)	0.92	−0.32(.13)	0.72*
Age	−0.09(.03)	0.91*	−0.09(.04)	0.91*
Male	−0.96(.54)	0.53	−0.59(.55)	0.55
Education	0.44(.31)	1.56	0.54(.36)	1.72
Information Internet Tasks	0.11(.24)	1.12	0.19(.27)	1.20
Operational Internet Tasks	0.06(.26)	1.06	0.16(.26)	1.17
Implication of Disclosure*TRDM	−	−	0.41(.16)	1.52*
Constant	0.95(2.27)	2.59	3.46(2.43)	32.01
Pseudo R square Log Likelihood	0.15 −43.82		0.21* −41.04	

The *R*^2^ value reported is the Nagerkerke *R*^2^.

*p < 0.05 (one-tailed).

As presented in Model 2, and in support of hypothesis 2, the interaction term (Implication of Disclosure * TRDM) was significant (*b* = 0.41, *p* = 0.005), demonstrating that thoughtfully reflective decision makers were more likely to deny access to the compromising requests when presented with the implication of disclosure. *Age* also remained statistically significant in this model (*b* = −0.09, *p* = 0.01).

Consistent with the findings presented for Study 1, being warned of the negative implications associated with not engaging in cyber hygiene did not increase cyber hygiene engagement. In fact, the introduction of the interaction term created an inverse (and significant) relationship between both *Implication of Disclosure* and *TRDM* (as separate variables) and *Deny Access*. Furthermore, in neither model were respondents’ *Gender*, *Education*, *Information Internet Tasks, nor Operational Internet Tasks* statistically significant.

**FIGURE 1:**
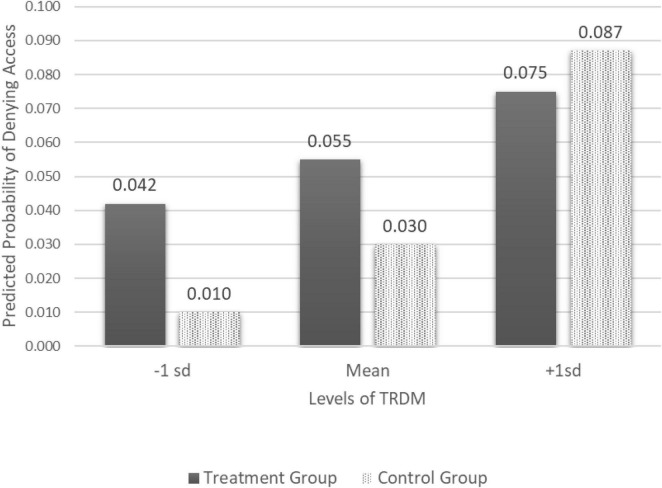
Predicted probabilities of denying access by level of TRDM across groups.

To better understand the interaction effect, we centered the *TRDM* scale to the mean and plotted the effect of *implication disclosure* on denied access to security requests across levels of *TRDM* (1 standard deviation above and below the mean). As indicated in Figure 1, participants who received the implication disclosure and scored 1 standard deviation above the mean in TRDM were more likely to deny access than those who scored 1 SD below the mean. Specifically, the probability increased from 0.042 to 0.075 (diff = 0.033, *p* < 0.05).

When comparing those who did not receive an implication disclosure, those who scored 1 SD above the mean were also more likely to deny access than those who scored 1 SD below the mean. Specifically, the probability increased from 0.01 to 0.087 (diff = 0.077, *p* > 0.05). However, unlike in the treatment group, this effect is not statistically significant.

Thus, these findings suggest that the interaction between *TRDM* and *Implication Disclosure* has an impact on security behaviors. Those with higher TRDM scores are more likely to be influenced by a warning message detailing the threats associated with poor security practices.

## Discussion

Tackling issues related to cybersecurity cannot be effectively accomplished by any singular academic discipline. Instead, a multidisciplinary approach is necessary for both theoretical and policy advancement (Howell and Burruss, 2020). In recent years, information security scholars have adopted various theoretical frameworks to explain security behaviors in cyberspace, but the efficacy of these frameworks is modest at best (Sommestad et al., 2015). For this reason, and since individuals have varying levels of cognitive-decision capabilities to accurately predict the overall costs and benefits (i.e., maximum utility) of their actions (Clarke and Cornish, 1985), the present study introduces a theoretical construct from the criminological literature that captures the essence of human agency (i.e., TRDM) to help explain engagement in target hardening behaviors (i.e., cyber hygiene).

Our findings are generally supportive of Paternoster and Pogarsky (2009) theory: cognitive decision-making capabilities are predictive of cyber hygiene engagement. More specifically, and in support of our first hypothesis, thoughtfully reflective decision makers are more likely to engage in computer privacy behaviors. In addition, and in support of our second hypothesis, thoughtfully reflective decision makers who are warned of the negative implications of not engaging in security behaviors are more likely to engage in computer security behaviors. However, thoughtfully reflective decision makers were not more likely to adopt security behaviors unless they were notified of the negative implications of not adopting the safeguards. Although thoughtfully reflective decision makers are, on average, better at making decisions that lead to quality outcomes (Paternoster et al., 2011), they are not necessarily more equipped with knowledge of computer systems and how to properly safeguard their networked devices from being compromised. Once they had more complete information, allowing them to make rational decisions, they did so.

Conversely, one does not need technical knowledge to understand the need to adopt privacy behaviors. Thus, thoughtfully reflective decision makers did not need additional information about the negative implications associated with not adopting privacy behaviors to adopt the safeguards. Another possible explanation for this observed effect is information overload, a state in which a person is overwhelmed by the amount of information presented for processing. Information overload is associated with decreased learning and poor decision-making (Buchanan and Kock, 2001; Zacharakis and Meyer, 2000). Since the public is, on average, more aware of threats to privacy (Bergström, 2015) the added information may trigger information overload and hinder participants’ ability to make privacy related decisions.

It is important to note that the educational intervention in this study focused solely on the “cost” aspect of decision-making, specifically the negative outcomes of granting privacy and security permissions to a malicious actor. This approach does not address the complexity involved in granting permission. Users often face ambiguous situations where the potential benefits of granting permissions, such as improved functionality or enhanced user experience, compete with perceived risks. This balancing act might lead to different decision-making dynamics than those captured by an intervention emphasizing only the negative aspects. Future research should explore whether the decision to grant permission is influenced by the user’s uncertainty about the legitimacy of the request. Nevertheless, irrespective of the cause, our findings provide support for TRDM in predicting target hardening behavior in the cyber environment and advance the theory by demonstrating how cognitive decision-making varies based on the complexity of the decision at hand.

Consistent with prior research (Cain et al., 2018), gender was not a significant predictor for security or privacy behaviors. However, older respondents were less likely to deny access to security requests. This finding may be a function of both age and the language of the messages. Older respondents may be more prone to cyber victimization, and less prone to engage in cyber hygiene behaviors, due to a lack of technical capabilities or understanding–particularly for requests involving technical configurations to firewalls, Javascript, etc. However, the frequency with which Internet tasks are engaged either did not matter or had the opposite effect than anticipated. Perhaps because the scales used in this study captured the frequency with which users engaged with technical and informational tasks rather than skills related to computer literacy. Future research should endeavor to better explore the relationship between age, computer literacy, and engagement in cyber hygiene behaviors. Finally, individuals with higher levels of education are also more likely to deny requests that infringe upon their privacy. More educated respondents may have been exposed to more information on the effects of privacy infringement or may be trained to think more critically about such requests.

### Implications

The findings discussed above are central to the development of theories capable of explaining behavioral patterns in the cyber-environment and evidence-based cybersecurity strategies. Prior research has demonstrated that cyber hygiene is synonymous with target hardening in the cyber-environment and reduces victimization experiences in a manner consistent with SCP (Howell, 2021). In finding that thoughtfully reflective decision makers are more likely to engage in target hardening practices, evidence is lent to the rational choice paradigm.

The rational choice paradigm offers practical solutions to crime reduction. If the decision-making calculus can be altered, through increased risks or rewards, persons can be nudged to make decisions that lead to more desirable outcomes. In other words, those who are less able to make quality decisions can be identified and nudged to engage in target hardening practices. The easiest solution to reduce cyber victimization, however, may be to take the decision to engage in these practices out of the users’ hands and instead automate cyber hygiene to the fullest extent possible. By eliminating the choice to engage in target hardening practices, and automating the process, those with low levels of TRDM will not be at increased susceptibility to victimization attempts.

A more difficult but long-lasting solution may be to educate about the dangers of poor cyber hygiene to increase engagement in protective behaviors for both thoughtfully reflective decision-makers and their counterparts. The findings presented above show the effect of interpersonal processes on the decision to engage in cyber hygiene and the ineffectiveness of a one-size-fits-all approach to cybersecurity. Those with reduced cognitive capabilities are less able to reflect on the implications of disclosure and, as a result, are less likely to engage in cyber hygiene behavior. Since TRDM is dynamic and changes over time, educational solutions related to cybersecurity should seek to evoke better decision making. This will not only improve cybersecurity but also promote better life outcomes.

### Limitations

The current study, although a novel way of testing the efficacy of TRDM, suffers from notable limitations. First, both field studies suffer from limited generalizability. Our sample was meant to be representative of the Israeli population. Israel has a diverse population with different backgrounds, resulting in a wide range of religious, ethnic, socio-economic, and cultural identities. Israel also has a unique age distribution wherein roughly 36% of Israel’s population is under the age of 20, while adults who are 65 years old and older comprise only 11% of the population. While these factors contribute to the importance of studying phenomena in Israel, differences exist between Israeli citizens who choose to participate in research studies and those who do not. Moreover, the Israeli population differs from other populations including, for example, the population of the United States. This issue is not unique to our study and can be remedied through replication using samples representative of other populations. Additionally, this study focuses on cell phone browsers to ensure consistent data collection. Future research should investigate if these results generalize to other devices and browsers.

Second, we chose to measure security and privacy separately, using a limited number of items. Future studies should further explore the most appropriate way to measure cyber hygiene as the online analogue of target hardening. This will allow for further development, and testing, of the SCP perspective in the cyber-environment.

Lastly, and most problematically, employing logistic regression modeling to test the experimental stimuli and TRDM may have introduced omitted variable bias into the study (Kamar et al., 2022). Indeed, we are unable to rule out alternative hypothesis. Future studies should consider implementing research designs that allow for the assessment of causality.

## Conclusion

Given these findings, future research in information security should no longer ignore human agency and cognitive functioning. More research on how an individual’s cognitive decision-making capabilities influence cybersecurity behavior, and examinations on how individuals can be nudged to make better decisions when confronted with threats (both in the real world and in cyberspace), is warranted. The study of cybercrime and cybersecurity related issues is inherently interdisciplinary with human, technical, political, and socioeconomic components. The development of theories capable of explaining behavioral patterns observable in the cyber-environment is not achievable by a singular academic discipline. The application of criminological theory to explain variation in information security behaviors serves as a modest step on the path toward interdisciplinary scholarship capable of advancing evidence-based solutions to (cyber)-crime reduction.

## Data Availability

The raw data supporting the conclusions of this article will be made available by the authors, without undue reservation.
